# A systematic review of taxane-containing regimens for metastatic breast cancer

**DOI:** 10.1038/sj.bjc.6602680

**Published:** 2005-07-19

**Authors:** D Ghersi, N Wilcken, R J Simes

**Affiliations:** 1 NHMRC Clinical Trials Centre, University of Sydney, Locked Bag 77, Camperdown, NSW 1450, Australia

**Keywords:** breast cancer, taxanes, systematic review

## Abstract

We compared the results of randomised trials comparing taxane-containing chemotherapy regimens with regimens not containing a taxane in women with metastatic breast cancer. The specialised register of the Cochrane Breast Cancer Group was searched in March 2004. Eligibility was assessed and data extracted from eligible studies by two reviewers. Hazard ratios (HR) were derived for time-to-event outcomes, and a fixed-effect model was used for meta-analysis. Tumour response rates were analysed as dichotomous variables. Of 21 eligible trials, 16 had published some results and 12 data on overall survival. An estimated 2621 deaths among 3643 women suggest a significant difference in overall survival in favour of taxane-containing regimens (HR 0.93, 95% confidence interval (CI) 0.86–1.00, *P*=0.05). The treatment effect on survival was similar if only trials of first-line chemotherapy were included, although not statistically significant. There appeared to be an advantage for taxanes in time to progression (HR 0.92, 95% CI 0.85–0.99, *P*=0.02) and overall response (odds ratio (OR) 1.34, 95% CI 1.18–1.52, *P*<0.001). There was significant heterogeneity across the trials (*P*<0.001), partly because of the varying efficacy of the comparator regimens. Taxane-containing regimens improved overall survival in women with metastatic breast cancer. Taxane-containing regimens are more effective than some, but not all, nontaxane-containing regimens.

Taxanes are among the most active chemotherapy agents used in the management of metastatic breast cancer. Paclitaxel was identified in 1971 as part of a National Cancer Institute (NCI) programme that screened medicinal plants for potential anticancer activity, and was first used in clinical trials in 1983 (Breast cancer: taxane clinical perspectives, 1996). Docetaxel was synthesised in 1986 and is similar to paclitaxel in its mechanism of action.

Initially, the use of taxanes was limited by hypersensitivity reactions, but once these were better managed (largely by premedication with steroids), taxane use became more frequent. Taxanes have become part of standard management in most western countries and are used as single agents or in combination with other chemotherapeutic drugs or the monoclonal antibody trastuzumab (Bernard-Marty *et al*, 2003).

The effect of taxanes on survival compared with other drugs or drug combinations is unclear. We therefore conducted a systematic review and meta-analysis to identify and synthesise the results of randomised clinical trials comparing taxane-containing chemotherapy regimens with regimens that did not contain a taxane. Prospectively, we asked the following questions:

(Q1): Regimen A plus taxane *vs* Regimen A (e.g. doxorubicin plus docetaxel *vs* doxorubicin alone)
(Q2): Regimen A plus taxane *vs* Regimen B (e.g. doxorubicin plus docetaxel *vs* doxorubicin plus cyclophosphamide)
(Q3): Single agent taxane *vs* Regimen C (e.g. docetaxel *vs* doxorubicin plus cyclophosphamide).

The planned outcome measures were survival (date randomised to date of death), time to progression (date randomised to date of progression or death), time to treatment failure, overall response, toxicity (specifically leukopenia, neurotoxicity, nausea or vomiting and treatment-related death) and quality of life.

## MATERIALS AND METHODS

Properly randomised controlled trials (i.e. where sequence generation and allocation concealment were adequate) comparing any regimen containing a taxane with any regimen not containing a taxane as first-line treatment for metastatic breast cancer were eligible. Trials that included both women with metastatic disease and women with isolated locoregional recurrent disease were eligible for inclusion if it was possible to distinguish between the two groups (data were reported separately) or if women with isolated locoregional recurrence were <20% of the total group. There were no age restrictions. Trials in which the primary intention was to investigate sequencing of treatment regimens were excluded.

The primary outcomes were survival and time to progression for which the hazard ratio (HR) is the most appropriate statistic. When possible, the HR and associated variances were extracted directly from the trial publication/s. If not reported, it was obtained indirectly using the methods described by Parmar *et al* (1998) using either other available summary statistics or from data extracted from published Kaplan–Meier curves. To allow for immature follow-up, the numbers at risk were adjusted based on estimated minimum and maximum follow-up times

A pooled HR was obtained from the derived observed (*O*)–expected (*E*) number of events and the variance for each trial using the fixed effect model (Yusuf *et al*, 1985). The pooled HR represents the overall risk of an event on taxane-containing chemotherapy *vs* nontaxane-containing chemotherapy. *χ*^2^ tests for heterogeneity were used to test for heterogeneity over all trials (see Alderson *et al*, 2003). *Post hoc* subgroup analyses were conducted for the type of taxane and prior exposure to anthracyclines. *χ*^2^ tests for interaction were applied to these subgroup analyses.

## RESULTS

On 12 March 2004, the search strategy in Table 1 was applied to the specialised register of trials maintained by the Cochrane Breast Cancer Group (see Search Strategy), resulting in the identification of 195 references to potentially eligible trials from the 7164 references on the register. We identified 21 eligible studies, of which three are ongoing (Table 2) (Dieras *et al*, 1995; Bishop *et al*, 1999; Chan *et al*, 1999; Nabholtz *et al*, 1999, 2003; Sjostrom *et al*, 1999; Luck *et al*, 2000; Paridaens *et al*, 2000; Bonneterre *et al*, 2001, 2002, 2003; Jassem *et al*, 2001; Zielinski *et al*, 2001; Biganzoli *et al*, 2002; Icli *et al*, 2002; Talbot *et al*, 2002; Bontenbal *et al*, 2003; Sledge *et al*, 2003; Goldhirsch, 2005; Heidemann, 2005). An additional two studies were identified but excluded: the status of one as a randomised trial was unclear (Gebbia *et al*, 2003), and the second randomised women to cease *vs* continue paclitaxel (Gennari *et al*, 2001). Not all trials reported on all outcomes. Some were less mature studies than others and had been reported in abstract form only.

Although the intention was to include only trials of first-line chemotherapy (i.e. no chemotherapy had been given except as adjuvant therapy), over half of the completed and published trials are of more-than-first-line therapy. All trials meeting the remaining eligibility criteria were therefore included in the review, and separate analyses were conducted for line of therapy. All trials eligible for Question 1 (adding a taxane to a chemotherapy regimen) and Question 2 (comparing any regimen containing a taxane with any regimen not containing a taxane) are of first-line chemotherapy.

It was not possible to assess accurately the quality of randomisation used in most studies owing to lack of information in the published articles. If the imbalance between treatment arms was deemed to be sufficient to lead to a suspicion of bias in the randomisation process, then this is reflected in the quality grade assigned to the randomisation process, details of which have been reported elsewhere (Ghersi *et al*, 2005).

Over 6300 women had been randomised to the 21 eligible trials, and time-to-event data for overall survival data were available for 57% of them. The data available for Question 3 (comparing a single-agent taxane with any regimen not containing a taxane) were more complete; nine of the 10 eligible trials (representing 2442 or 84% of the 2780 women randomised to this question) had reported overall survival data.

One study was a three-armed trial eligible for both Questions 1 and 3 (Sledge *et al*, 2003). This was taken into account when the overall effect of taxanes was calculated (by halving the control group).

### Overall survival

The trials of first-line chemotherapy suggest that there may be a benefit in terms of overall survival in favour of taxanes, with an HR of 0.92 (95% confidence interval (CI) 0.84–1.02, *P*=0.11). When all trials are included, there is a statistically significant difference of similar magnitude in favour of taxane-containing regimens, with an HR of 0.93 (95% CI 0.86–1.00, *P*=0.05). There was no statistically significant heterogeneity across the trials.

**Figure 1:**
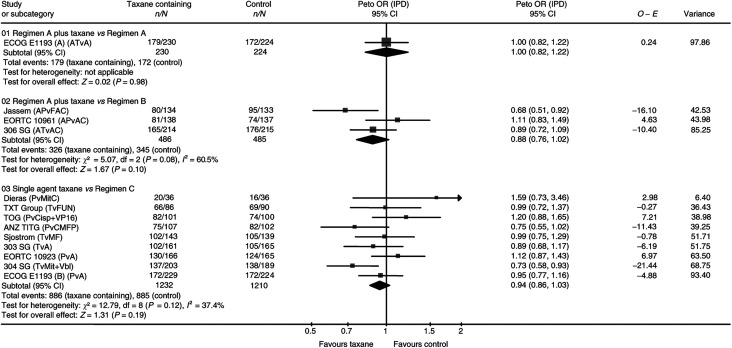
Overall survival. Overall HR for overall survival is 0.93, 95% CI 0.86–1.00, *P*=0.05. Test for heterogeneity: χ^2^=18.58, df=12 (*P*=0.10), *I*^2^=35.4%.

The results for those individual trials that reported on survival are shown in Figure 1. Only three of the nine studies eligible for Question 2 (comparing any regimen containing a taxane with any regimen not containing a taxane) provided information on survival, representing 35% of the estimated number of patients. The three trials suggested that there might be a benefit in terms of overall survival in favour of taxanes (HR 0.88, 95% CI 0.76–1.02, *P*=0.10), but this was not statistically significant.

Nine of the 10 studies eligible for Question 3 (comparing a single-agent taxane with any regimen not containing a taxane) reported on overall survival, which suggests a benefit in favour of the taxane arm (HR 0.94, 95% CI 0.86–1.03, *P*=0.19) with no statistically significant heterogeneity. A similar but nonsignificant effect was seen if trials with potentially suboptimal comparators (those comparing taxane with mitomycin±vinblastine, and fluorouracil with vinorelbine) (Dieras *et al*, 1995; Nabholtz *et al*, 1999; Bonneterre *et al*, 2002) were excluded (HR 0.97, 95% CI 0.88–1.08, *P*=0.62), or if the analysis of this subgroup was limited to trials of first-line chemotherapy (HR 0.95, 95% CI 0.83–1.10, *P*=0.50).

### Time to progression

Trials were not consistent in the way they defined this outcome. Trials that started the clock at the time of randomisation were included. Details of the definition used for this outcome for each trial has been reported elsewhere (Ghersi *et al*, 2005).

**Figure 2:**
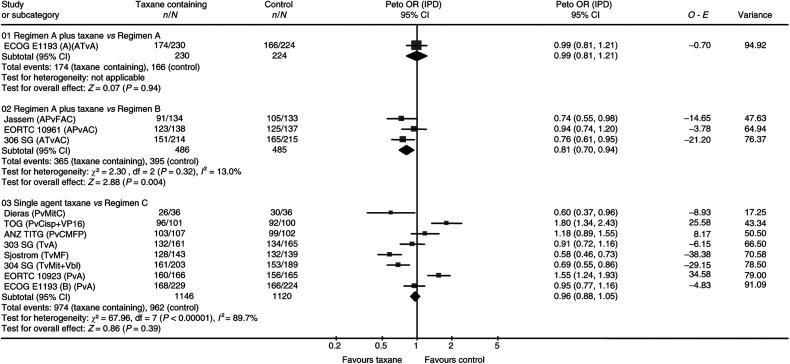
Time to progression. Overall HR for time to progression is 0.92, 95% CI 0.85–0.99, *P*=0.02. Test for heterogeneity: χ^2^=74.41, df=11 (*P*<0.00001), *I*^2^-85.2%.

The six trials of first-line chemotherapy suggest that there is no detectable difference between taxane and nontaxane-containing regimens (HR 0.99, 95% CI 0.90–1.09, *P*=0.88). If data from all 11 of the 21 eligible trials reporting this outcome are included, there is a statistically significant benefit in favour or taxanes (HR 0.92, 95% CI 0.85–0.99, *P*=0.02) (Figure 2). There was, however, significant heterogeneity across trials for all time-to-progression analyses (*P*<0.00001, *I*^2^=89.7%).

Only three of the 21 eligible trials reported data on time to treatment failure. It was therefore not considered appropriate to pool data across trials.

### Overall response rates

**Figure 3:**
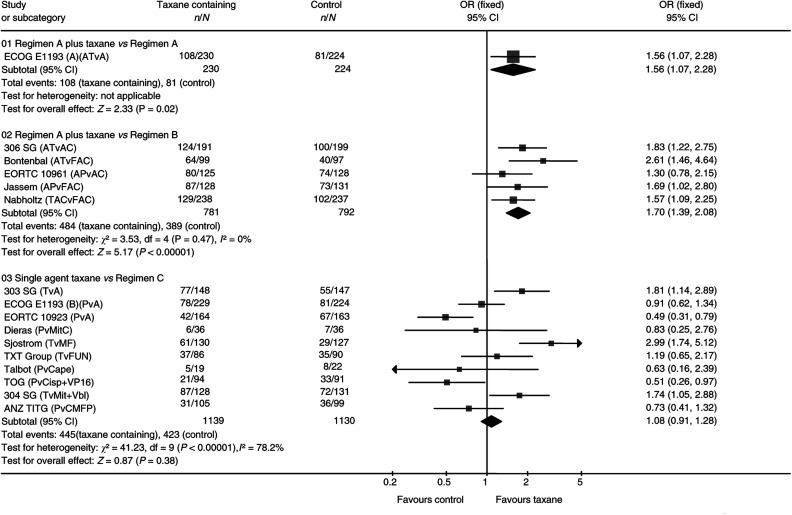
Overall response. Overall OR for overall response is 1.34, 95% CI 1.18–1.52, *P*<0.0001. Test for heterogeneity: χ^2^=55.41, df=15 (*P*<0.00001), *I*^2^=72.9%.

Sufficient data from 15 of the 21 eligible trials were available to enable an odds ratio (OR) for response rates to be calculated. There were some differences in the definition of response across (but not within) trials. The analysis of the 2787 assessable patients in first-line trials indicate a statistically significant difference in favour of taxane-containing regimens (OR 1.28, 95% CI 1.10–1.50, *P*=0.002). This difference remained when all trials reporting this outcome were included (OR 1.34, 95% CI 1.18–1.52, *P*<0.0001). There was significant heterogeneity across trials for this outcome (*P*<0.0001) (Figure 3).

### Toxicity

Four of the nine studies eligible for Question 2 (comparing any regimen containing a taxane with any regimen not containing a taxane) reported on toxicity (Table 3). Taxanes were associated with significantly more leukopenia and neurotoxicity, but less nausea and vomiting in assessable patients. Of those eligible for Question 3 (comparing a single-agent taxane with any regimen not containing a taxane), seven studies reported on leukopenia, nine on nausea or vomiting and neurotoxicity and four on hair loss (Table 4). Taxanes were associated with significantly worse neurotoxicity and hair loss, but less leukopenia and nausea or vomiting.

### Quality of life

In all, 10 trials had collected quality-of-life data, eight of which had reported results (Bishop *et al*, 1999; Chan *et al*, 1999; Nabholtz *et al*, 1999, 2003; Hakamies-Blomqvist *et al*, 2000; Kramer *et al*, 2000; Carmichael, 2001; Jassem *et al*, 2001; Biganzoli *et al*, 2002; Sledge *et al*, 2003). The type of instrument used and the way in which quality of life was reported varied across trials, as did the completion rate by patients of quality-of-life instruments. Some studies reported problems with patients in poorer health not completing questionnaires (e.g. Nabholtz *et al*, 1999). For these reasons, it was decided not to statistically pool quality-of-life data. None of the individual trials reported a statistically significant difference in overall quality of life, or in any of the subscales, between taxane- and nontaxane-containing chemotherapy regimens.

### Treatment-related death

The trials reported 54 treatment-related deaths: 24 on taxane-containing regimens and 30 on the nontaxane-containing regimens. There was no statistically significant difference between the two groups (OR 0.80, 95% CI 0.48–1.32, *P*=0.41).

### Subgroup analyses

#### Single-agent taxane compared with single-agent anthracycline

The three trials comparing single-agent taxane with single-agent anthracycline (an estimated 916 events in 1110 women) showed no detectable difference in time to progression (HR 1.10, 95% CI 0.97–1.26, *P*=0.12) with some evidence of heterogeneity (*P*=0.001). An estimated 812 deaths in 1110 women showed no detectable difference in overall survival (HR 1.00, 95% CI 0.88–1.15, *P*=0.94) and no statistically significant heterogeneity.

#### Single-agent taxane compared with nonanthracycline-containing combinations

The six trials comparing single-agent taxane with a nonanthracycline-containing combination (estimated 966 deaths in 1332 women) favoured taxane-containing regimens for overall survival (HR 0.91, 95% CI 0.80–1.03, *P*=0.13) with no statistically significant heterogeneity. For time to progression, the five studies with usable data (1020 events in 1156 women) favoured taxane-containing regimens (HR 0.85, 95% CI 0.75–0.96, *P*<0.0008), with statistically significant heterogeneity (*P*<0.0001, *I*^2^ 91.3%).

#### Type of taxane

*Post hoc* subgroup analyses were conducted to investigate the treatment effect within the types of taxane. Data from the 2038 women randomised to seven trials using paclitaxel show no detectable difference in overall survival (HR 0.97, 95% CI 0.87–1.07, *P*=0.54) or in time to progression. Data from the 1605 women randomised to five trials using docetaxel showed a statistically significant difference in overall survival and time to progression in favour of the taxane-containing regimen (HR for overall survival 0.88, 95% CI 0.78–0.98, *P*=0.02) with no statistically significant heterogeneity.

**Figure 4:**
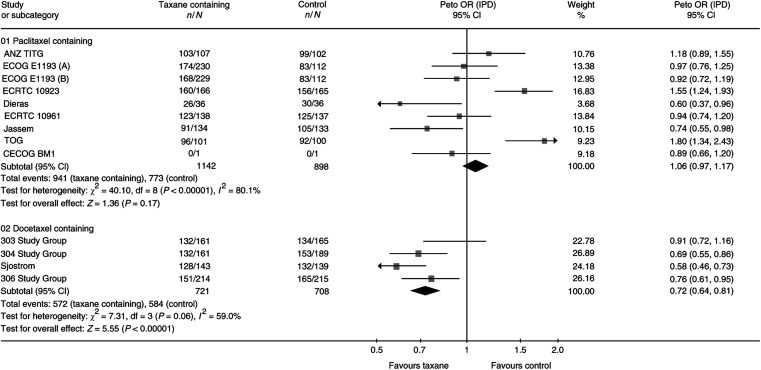
Time to progression for subgroup type of taxane.

There was a statistically significant difference in time to progression between women who had received docetaxel compared to those who had received paclitaxel (test for interaction *P*<0.001) (Figure 4). The interpretation of this result is complicated by the significant heterogeneity in both the docetaxel and paclitaxel trials and may relate to the choice of comparator in these trials.

#### Previous exposure to anthracyclines

**Figure 5:**
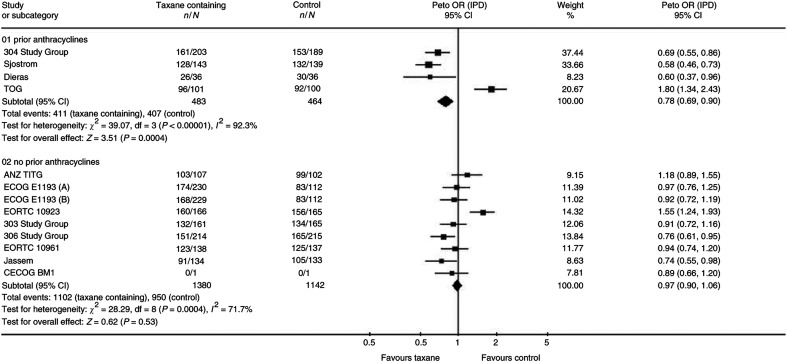
Time to progression for subgroup previous anthracycline.

*Post hoc* subgroup analyses were also used to investigate the treatment effect in patients who had or had not received previous anthracyclines. Data from the 1123 women randomised to the five trials in women who had received anthracyclines favoured taxane-containing regimens in terms of overall survival (HR 0.94, 95% CI 0.82–1.08, *P*=0.39) and time to progression. There was no detectable difference for either outcome in anthracycline-naive women, although there was a significant difference in favour of taxanes for overall response (OR 1.21, 95% CI 1.03–1.43, *P*=0.02). There was statistically significant difference in time to progression (*P*=0.006) between women who had received prior anthracyclines and those who had not (*P*<0.001) (Figure 5).

## DISCUSSION

Despite the relative immaturity of many of the studies included in this review, there is sufficient evidence to conclude that on average, taxane-containing regimens are associated with a statistically significant improvement in overall survival compared with nontaxane-containing regimens. This is consistent with emerging data from trials employing taxanes in the adjuvant setting (Nowak *et al*, 2004).

Conclusions about the effects of taxanes on other end points (such as response rate and time to progression), the effects of taxanes in various subgroups and the differential effects of paclitaxel and docetaxel are of clinical interest, but are statistically less secure. Taxane-containing regimens were associated with more leukopenia and neurotoxicity, but less nausea and vomiting, than the control group, and the effect on quality of life did not appear to differ in any of the trials.

At the time of this review, overall survival data were available for only 12 of the 20 eligible trials. This may relate to the relative immaturity of some of the trials, or reporting bias (specifically the tendency to report positive results early) may exist. There may also be unpublished trials that were not identified in our search. The treatment effects reported may therefore be overestimated.

The initial eligibility criteria for this review limited trials to those comparing taxane- with nontaxane-containing regimens as first-line chemotherapy. A decision was made to include comparisons of more-than-first-line chemotherapy owing to the limited number of completed trials (most of the first-line trials had not reported survival data). Results for overall survival and time to progression limited to the available first-line treatment trials suggest a benefit in favour of taxane-containing regimens, but this is not statistically significant. Nevertheless, the observed results are consistent with those based on all trials and may be due to lack of statistical power.

Some heterogeneity across the trials is to be expected given the different drugs, dosages and schedules being used and the different patient groups and treatment settings. There is, however, strong statistical evidence of heterogeneity among the trials in the effect of treatment on time to progression and response (*P*<0.00001), and one explanation for this is the varying efficacy of the comparator regimens. For example, the regimens of mitomycin±vinblastine, and fluorouracil+vinorelbine could be regarded as suboptimal chemotherapy for breast cancer. If these regimens are excluded, the advantages for a single-agent taxane, when compared with a nontaxane-containing regimen, are no longer statistically significant. While opinions will vary regarding those regimens that could be considered to be suboptimal, it is reasonable to conclude that taxanes are more effective than some, but not all, regimens with which they have been compared, and are at least as effective as the other regimens.

The analyses of most relevance to clinical practice are comparisons of the different taxanes, and the contexts in which they are used (i.e. in anthracycline-naïve patients or not). The available data suggest that docetaxel may be more active than paclitaxel, at least when given in 3-weekly schedules. This is based on an indirect comparison of these two drugs in trials with statistical heterogeneity, but is consistent with the preliminary results of a trial directly comparing the taxanes (Jones *et al*, 2003).

Furthermore, weekly schedules of taxanes are now commonly used and may have a different efficacy-to-toxicity ratio. Ongoing trials, in all stages of breast cancer, are investigating the relative efficacy of different taxanes and different schedules of those taxanes. The benefit of taxanes also appears to be less apparent in patients who have not had previous anthracyclines. While subset analyses may be useful for informing clinical practice, interpreting such analyses requires caution, given the smaller number of patients in each subgroup, and the potential effect of confounding.

This review includes data from 12 studies (3643 randomised women) reporting time-to-event outcomes, and 16 studies (4287 randomised women) reporting response as an outcome. When complete, the data from all 21 eligible studies will contribute information on over 6000 randomised women to future updates of this review.

This paper is based on a Cochrane review published in the Cochrane Library 2004, Issue 3 (see www.CochraneLibrary.net for information). Cochrane reviews are regularly updated as new evidence emerges and in response to comments and criticisms, and the Cochrane Library should be consulted for the current version of the review.

## Figures and Tables

**Table 1:** Search strategy

The specialised register is based on a detailed search strategy consisting of numerous MeSH headings and text word combinations. This strategy is applied routinely to the Medline and Embase databases. Similar (although less detailed) searches are conducted of the PDQ database and the Cochrane Central Register of Controlled Trials. The major relevant conference proceedings are also searched (either by hand or electronically) and included in the register. For further details, please see the module of the Cochrane Breast Cancer Group published on the Cochrane Library (Simes *et al*, 2005).
References on the register are coded to facilitate searching. The codes ‘advanced’ and ‘chemotherapy’ were applied to the specialised register and combined with the Keywords (imported with the references from Medline) ‘Taxol’, ‘docetaxel’ or ‘paclitaxel’, and a search of all nonindexed fields for the following text words: taxane, taxanes, taxol, taxotere, paclitaxel, paxene, nsc-125973, docetaxel or anzatax.
The reference lists of publications of eligible studies and related literature reviews were also searched.

**Table 2:** Studies included in the review

**Trial ID**	**Summary details**	**Arm 1: taxane containing** a	**Arm 2: control**	**Majority first line for MBC**	**Majority anthracycline naïve**	**Accrual**
*Regimen A plus taxane vs Regimen A*						
ECOG E1193 (A) (Sledge *et al*, 2003)	Open: 1993–1995	AT (paclitaxel+doxorubicin+G-CSF)	Doxorubicin	Y	Y	739
EU-93011 (Heidemann, 2005)	Open: 1994–(ongoing)	Docetaxel+mitoxantrone	mitoxantrone	Y	Both	300
SAKK (Goldhirsch, 2005)	Open: unk (ongoing)	Paclitaxel+trastuzumab	trastuzumab	Y	Both	170–250
						
*Regimen A plus taxane vs Regimen B*						
306 Study Group (Nabholtz *et al*, 2003)	Open: 1996–1998	AT (docetaxel+doxorubicin)	AC (cyclophosphamide+ doxorubicin)	Y	Y	429
Bontenbal (Bontenbal *et al*, 2003)	Open: 1997–2002. Abstract only	AT (docetaxel+doxorubicin)	FAC (fluorouracil+doxorubicin+ cyclophosphamide)	Y	Y	216
Nabholtz (Nabholtz *et al*, 2001)	Open: 1998–1999. Abstract only	TAC (docetaxel+doxorubicin+ cyclophosphamide)	FAC (fluorouracil+doxorubicin+ cyclophosphamide)	Y	N	484
EORTC 10961 (Biganzoli *et al*, 2002)	Open: 1996–1999	AT (paclitaxel+doxorubicin)	AC (cyclophosphamide+ doxorubicin)	Y	Y	275
Jassem (Jassem *et al*, 2001)	Open: 1996–1998	AT (paclitaxel+doxorubicin)	FAC (fluorouracil+doxorubicin+ cyclophosphamide)	Y	Y	267
*AGO* (Luck *et al*, 2000)	Open: 1996–1999. Abstract only	ET (paclitaxel+epirubicin)	EC (cyclophosphamide+epirubicin)	Y	Y	*505*
Bonneterre (Bonneterre *et al*, 2001)	Open: 1998–2000. Abstract only	ET (docetaxel+epirubicin)	FEC (fluorouracil+epirubicin+ cyclophosphamide)	Y	Both	141
UKCCCR AB01 (Carmichael, 2001)	Open: 1996–1999. Abstract only.	ET (paclitaxel+epirubicin)	EC (cyclophosphamide+epirubicin)	Y	Y	705
CECOG BM1 (Zielinski *et al*, 2001)	Open: 1999– (ongoing)	GET (paclitaxel, epirubicin, gemcitabine)	FEC (fluorouracil, epirubicin, cyclophosphamide)	Y	?	Sample size unknown
						
*Single agent taxane vs Regimen C*						
303 Study Group (Chan *et al*, 1999)	Open: 1994–1997	Docetaxel	Doxorubicin	N	Y	326
304 Study Group (Nabholtz *et al*, 1999)	Open: 1994–1997	Docetaxel	Mitomycin+vinblastine	N	N	392
Sjostrom (Sjostrom *et al*, 1999)	Open: 1994–1997	Docetaxel	MF (methotrexate+fluorouracil)	N	N	283
TXT Group (Bonneterre *et al*, 2002)	Open: 1995–1997	Docetaxel	FUN (fluorouracil+vinorelbine)	N	N	176
ANZ TITG (Bishop *et al*, 1999)	Open: 1993–1995	Paclitaxel	CMFP (cyclophosphamide+ methotrexate+fluorouracil+ prednisone)	Y	Y	209
Dieras (Dieras *et al*, 1999)	Open: unk	Paclitaxel	Mitomycin	N	N	81
ECOG E1193 (B) (Sledge *et al*, 2003)	Open:1993–199	Paclitaxel	Doxorubicin	Y	Y	739
EORTC 10923 (Paridaens *et al*, 2000)	Open: 1993–1996	Paclitaxel	Doxorubicin	Y	Y	331
Talbot (Talbot *et al*, 2002)	Open: 1996–1997	Paclitaxel	Capecitabine	N	N	42
TOG (Icli *et al*, 2002)	Open: 1997–2002. Abstract only	Paclitaxel	Cisplatin+VP-16	N	N	201

MBC=metastatic breast cancer.

a All taxane-containing regimens were 3-weekly cycles.

**Table 3:** Acute toxicity, grades III and IV combined: Regimen A+taxane *vs* Regimen B

**Site of toxicity**	**Number of trials**	**Taxane events/patients**	**Control events/patients**	**OR (95% CI)**
*Assessable patients*				
Leukopenia^a^	4 (a, b, c, d)	538/591	470/585	2.48 (1.75–3.52)
Nausea or vomiting^b^	3 (a, b, c)	32/472	59/471	0.51 (0.32–0.80)
Neurotoxicity	3 (a, b, c)	20/466	0/469	43.11 (2.60–714.94)
				
Randomised patients				
Leukopenia^a^	4 (a, b, c, d)	538/595	470/591	2.43 (1.73–3.41)
Nausea or vomiting^b^	3 (a, b, c)	32/487	59/484	0.51 (0.32–0.79)
Neurotoxicity	3 (a, b, c)	20/487	0/484	42.49 (2.56–704.56)

OR=odds ratio; CI=confidence interval.

a=EORTC 10961; b=Jassem; c=306 Study Group, d=Bontenbal.

*Note*: Jassem reported 264 of the 267 patients received treatment but did not report denominator for each treatment arm. Assumed % of randomised patients.

a Data on grade III or IV neutropenia was included if data on leukopenia was not reported.

b If data on nausea and vomiting were reported separately, data on vomiting was included.

**Table 4:** Acute toxicity, grades III and IV combined: single-agent taxane *vs* Regimen C

**Site of toxicity**	**Number of trials**	**Taxane events/patients**	**Control events/patients**	**OR (95% CI)**
*Assessable patients*				
Leukopenia	7 (a, b, c, d, g, h, i)	334/655	422/663	0.59 (0.48–0.74)
Nausea or vomiting	9 (a, b, c, d, e, f, g, h, i)	30/1007	92/1000	0.30 (0.20–0.46)
Neurotoxicity	9 (a, b, c, d, e, f, g, h, i)	63/1007	10/1000	6.61 (3.37–12.95)
Hair loss	4 (a, h, i)	119/210	31/211	7.59 (4.75–12.13)
				
Randomised patients				
Leukopenia	7 (a, b, c, d, g, h, i)	334/682	422/684	0.60 (0.48–0.74)
Nausea or vomiting	9 (a, b, c, d, e, f, g, h, i)	30/1028	92/1012	0.30 (0.20–0.46)
Neurotoxicity	9 (a, b, c, d, e, f, g, h, i)	63/1028	10/1012	6.54 (3.34–12.82)
Hair loss	4 (a, h, i)	119/213	31/214	7.47 (4.68–11.92)

OR=odds ratio; CI=confidence interval.

a=ANZ TITG; b=Chan; c=Dieras; d=EORTC 10923; e=Nabholtz 1; f=Sjostrom; g=TOG; h=TXT Study Group; i=Talbot.

*Note*: ANZ TITG and TXT Study Group both graded hair loss using the WHO criteria, and Talbot used the NCIC Common Toxicity Criteria.
